# Gut microbiota composition in relation to intake of added sugar, sugar-sweetened beverages and artificially sweetened beverages in the Malmö Offspring Study

**DOI:** 10.1007/s00394-020-02392-0

**Published:** 2020-10-08

**Authors:** Stina Ramne, Louise Brunkwall, Ulrika Ericson, Nicola Gray, Gunter G. C. Kuhnle, Peter M. Nilsson, Marju Orho-Melander, Emily Sonestedt

**Affiliations:** 1 Department of Clinical Sciences Malmö, Faculty of Medicine, Lund University, Malmö, Sweden; 2 Center of Computational and Systems Medicine, Australian National Phenome Centre, Murdoch University, Murdoch, Australia; 3 Department of Food and Nutritional Sciences, School of Chemistry, Food and Pharmacy, University of Reading, Reading, UK

**Keywords:** Gut microbiota, Added sugar, Urinary sugars biomarker, Sugar-sweetened beverages, Artificially sweetened beverages

## Abstract

**Purpose:**

It has been suggested that a high intake of sugar or sweeteners may result in an unfavorable microbiota composition; however, evidence is lacking. Hence, in this exploratory epidemiological study, we aim to examine if intake of added sugar, sugar-sweetened beverages (SSBs) or artificially sweetened beverages (ASBs) associate with the gut microbiota composition.

**Methods:**

Participants (18–70 years) in the Malmö Offspring Study have provided blood, urine, and fecal samples and completed both web-based 4 day food records and short food frequency questionnaires. The gut microbiota was assessed by 16S rRNA sequencing, processed in QIIME and matched to Greengenes (v.13.8), giving 64 included genera after filtering. Intake of added sugar (*n* = 1371) (also supported by the overnight urinary sugar biomarker in a subgroup *n* = 577), SSBs (*n* = 1086) and ASBs (*n* = 1085) were examined as exposures in negative binomial regressions.

**Results:**

Various genera nominally associated with intake of added sugar, SSBs, and ASBs. Only the negative association between SSB intake and *Lachnobacterium* remained significant after multiple testing correction. A positive association between SSB intake and the Firmicutes:Bacteroidetes ratio was also observed.

**Conclusion:**

In this wide population, the cross-sectional associations between added sugar and sweet beverage intake and the gut microbiota are modest, but the results suggest that SSB intake is associated negatively with the genus *Lachnobacterium* and positively with the Firmicutes:Bacteroidetes ratio. Larger studies, preferably using metagenomic sequencing, are needed to further evaluate if a link exists between intake of sugars and sweeteners and the human gut microbiota.

**Electronic supplementary material:**

The online version of this article (10.1007/s00394-020-02392-0) contains supplementary material, which is available to authorized users.

## Introduction

In addition to added sugar consumption, both consumption of sugar-sweetened and artificially sweetened beverages have been associated with increased mortality [1, 2], as well as obesity and cardiometabolic disease [3, 4]. Recent progress within the research field of the gut microbiome also suggests the microbiota to importantly contribute to obesity and cardiometabolic disease [5]. It has been proposed that a high intake of sugar and low-calorie/nonnutritive sweeteners results in an unfavorable microbiota composition [6, 7]. The scientific evidence for this is however lacking. The few existing findings on the effect of intake of sugar and some low-calorie/nonnutritive sweeteners on the gut microbiota composition are mainly provided by small animal studies [8].

The potential pathways of how sugar intake might affect the gut microbiota is not clear since sugars theoretically do not reach the colon as their absorption takes place in the small intestine. However, high intake of sugars has been shown to cause alterations in the gut microbiota in rodents [9, 10]. In addition, there is accumulating research showing that absorption of fructose can be quite inefficient in humans, especially when large amounts of fructose are consumed within a short time period (i.e. how candy and sugar-sweetened beverages typically are consumed) and even more so when fructose is unbound (i.e. as in products sweetened with high-fructose corn syrup) [11, 12]. Hence, an increased amount of fructose reaches the colon, which is suggested to contribute to a gut microbiota composition that associates with obesity and metabolic disease [6]. In addition, a recent study has observed how sugar intake can suppress the BT3172 gene, a colonization factor for *Bacteroides thetaiotaomicron*, hence inhibiting colonization of this bacteria in the gut of mice [13]. This poses an additional potential mechanism for how a high sugar intake might affect the gut microbiota. Furthermore, another potential link could be induced by lack of e.g. fibers [14] and flavonoids [15], as often is seen in a typical high sugar diet [16].

Consumption of the artificial sweeteners sucralose and saccharine could potentially shift the microbiota composition towards a dysbiosis as seen in rodents [8]. For saccharin, this dysbiosis could induce glucose intolerance, which also was observed in a small human trial [17]. However, saccharin is only one of many different low-calorie/nonnutritive sweeteners. Consumption of low-calorie/nonnutritive sweeteners in the form of certain sugar alcohols might even serve as prebiotics and could potentially enrich the microbiota composition, while other sugar alcohols might not have those beneficial properties [8].

In a recent review, Di Rienzi et al. summarized three main potential pathways on how our gut microbiome may adapt to high intake of sugar or sweeteners; compositional changes, transcriptional changes and genetic changes that can cause variations within the bacterial strains, all with the purpose to adjust to better utilize the present substrates [7]. However, there is a shortage of human studies on this topic. Consequently, the link between intake of added sugars, sugar-sweetened beverages (SSBs), and artificially sweetened beverages (ASBs) and the gut microbiota composition need to be studied in large free-living human populations to understand their impact on human health and the health of our inhabitant bacteria. In this exploratory epidemiological study, we aim to examine if intake of added sugar, SSBs or ASBs is associated with any bacterial genera or measures of microbiota diversity.

## Method

### Subjects

Adult children and grandchildren of participants in the Malmö Diet and Cancer-Cardiovascular Cohort were recruited to participate in the prospective Malmö Offspring Study (MOS) from 2013 and onwards. Baseline data, including biological samples, questionnaire data and dietary assessment, were collected during two visits to the research clinic. In this cross-sectional analysis of MOS participants recruited until the end of April 2017 (*n* = 2644), we included the 1371 participants with complete data from 4 day food records (4DFR) (853 excluded) and 16S rRNA sequencing (261 excluded) that were free from diabetes (43 excluded), had a reported energy intake within 500–6000 kcal/day (3 excluded) and did not have any missing data on model covariates (113 excluded). All the participants signed a written informed consent when entering. MOS was granted ethical approval by the Regional Ethics Committee in Lund (Dnr.2012/594) and have thus been performed in accordance with the Declaration of Helsinki.

### Microbiome sequencing and taxonomic classification

During the first visit to the research clinic, participants received thorough instructions how to collect a fecal sample at home. Instructions were provided to store the sample in the freezer until return to the second visit at the research clinic, where the samples were frozen at − 80 °C. Extraction of bacterial DNA was performed using the QIAmp column stool kit and 16S rRNA (V1-V3 region) was sequenced with HiSeq Illumna at GATC Biotech (Germany). A total of 937 892 146 reads were included for downstream analysis, with an average of 434 008 reads per individual. Sequences were binned into operational taxonomic units using QIIME (1.9.1) and matched with the Greengenes database (v.13.8). From Greengenes, data was extracted on genus level and a total of 542 genera were identified. We filtered the data by removing genera identified in less than 3 participants and/or with a relative abundance of less than 0.01%, resulting in 64 included genera. Some genera are presented within hard brackets which indicate a proposed taxonomy by the Greengenes database. The absolute abundances of these bacterial genera were normalized through cumulative sum scaling using MetagenomicSeq in R. For calculation of the Firmicutes:Bacteroidetes ratio, the relative abundances of these two phyla were used. This ratio was highly skewed and therefore log transformed for statistical analyses.

### Dietary assessment method

On the day after the first visit to the research clinic, participants began recording everything consumed for 4 days prospectively. The participants entered their consumption data into the web-based 4DFR method Riksmaten2010, developed by the Swedish National Food Agency. Added sugar intake was estimated from total intake of monosaccharides and sucrose, subtracting for the amount naturally occurring in fruit, vegetables and fruit juices, as previously described in detail [18]. Added sugar intake as a percentage of non-alcohol energy intake (E%) from 4DFR was grouped into three groups < 10E%, 10-20E% and > 20 E%. We studied intake of added sugar rather than total sugar because added sugar intake is what is believed to primarily be important for cardiometabolic risk. Reported intakes of SSB and ASB from the 4DFR were also grouped into three groups 0 ml/day, > 0–100 ml/day and > 100 ml/day. ASBs may include beverages that are sweetened with both artificial or natural low-calorie or nonnutritive sweeteners. Fiber intake was evaluated as fiber density in g/1000 kcal.

Participants also filled in a short food frequency questionnaire (FFQ) covering the past 6 months. Consumption frequencies addressing SSB and ASB intakes ranged from never/seldom to several times/day on an 8-level scale. The reported SSB and ASB intakes from the FFQ were grouped to match the amounts of the three groups of the 4DFR-reported intake of SSBs and ASBs, assuming that a serving size of SSB and ASB are 250 ml, accordingly: never/seldom (0 ml/day), 1 time/month to 1–2 times/week (> 0–71 ml/day), and 3–4 times/week to several times/day (> 107 ml/day).

**Fig. 1:**
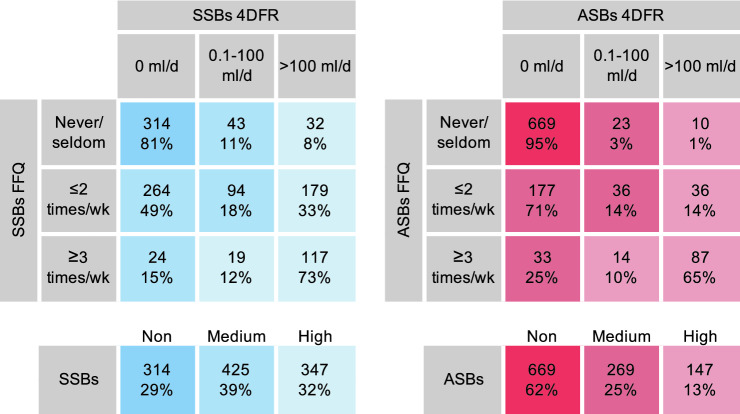
Combining and grouping of the SSB and ASB variables from 4DFR and FFQ into non-consumers, medium consumers and high consumers

Intake data of SSBs and ASBs from the 4DFR and FFQ were combined by crosstabulation of the three groups. The 4DFR was the dominant method since it directly reflects the same time period as the collected microbiota data and the FFQ was used to correct for the 4DFR’s limited ability to capture habitual intake of foods consumed irregularly. Those reporting non-consumption using both methods were grouped as certain non-consumers. Those reporting 0 ml/day from the 4DFR but did not report non-consumption in the FFQ, plus those reporting > 0–100 ml/day from the 4DFR but ≤ 2 times/week in the FFQ were grouped into medium consumers. Lastly, everyone reporting > 100 ml/day from the 4DFR, plus those reporting > 0–100 ml/day from the 4DFR but ≥ 3 times/week in the FFQ were group into high consumers. The grouping procedure is visualized in Fig. 1.

### Overnight urinary sugars

Overnight urine samples were collected on the morning of the second research visit, including any urine excreted during the night before the visit. Urine samples were stored for a maximum of 4 h in fridge at the research clinic before aliquoted and frozen at – 80 °C. In a subsample, urinary sucrose and fructose concentrations were measured using liquid chromatography–tandem mass spectrometry as described previously [18]. The urinary sucrose and fructose concentrations were divided by urine osmolality to adjust for urine dilution and further investigated as their sum (U-sugars). The reported added sugar intake from 4DFR was combined with the U-sugars for improved added sugar intake assessment using their first principle component (PC) according to Freedman et al. [18, 19]. The urinary fructose measurement was further used to find potential malabsorbers of fructose. If fructose is malabsorbed, it does not reach the circulation and consequently not the urine either. Using crosstabulation of quartiles, those reporting high added sugar intake but low urinary fructose were categorized as potential fructose malabsorbers (highest or second highest of added sugar, while lowest of urinary fructose, or highest of added sugar while lowest or second lowest of urinary sugars) as seen in Supplemental Fig. 1.

### Data on confounding factors

At the research clinic, weight was measured wearing light clothing and height was measured using a stadiometer. Body mass index (BMI) was calculated as weight (kg)/height^2^ (m). Waist circumference was measured in a standing position. Participants were instructed on how to fill out a lifestyle questionnaire which included smoking, drinking, and physical activity habits, as well as drug use, family history of diseases, and socioeconomic factors. Smoking habits were categorized as never smoked, ex-smoker, irregular smoker and regular smoker, and alcohol habits were categorized from a combination of the lifestyle questionnaire and the reported alcohol intake from the 4DFR as described previously [20]. The following yes–no question was included in the lifestyle questionnaire addressing antibiotics: Have you taken any antibiotics the past 6 months? The FFQ included the following question addressing probiotics, with answers on an 8-level scale ranging from never/seldom to several times/day: How often do you eat probiotics (in fruit drinks, dairy products or as pills)? The physical activity level (PAL) values were obtained from combination of two questions incorporated in the web-based 4DFR, one regarding physical activity at work and the other regarding leisure-time physical activity.

### Statistical analysis

Statistical analysis was mainly performed in StataSE 15.0 (StataCorp, USA). Baseline characteristics were compared in the lowest and highest of the three intake groups of added sugar, composite PC of added sugar and U-sugars, and the combined measures of 4DFR-reported and FFQ-reported intake of both SSB and ASB intake, using *t* tests for continuous variables and Chi-square tests from categorical variables.

Multivariable negative binomial regression analysis was performed to evaluate the associations between the four different exposure variables categorized into three intake groups and 64 bacterial genera. Adjusted means of normalized abundances were calculated in the three intake groups of exposure. Adjustment for multiple testing was performed through false discovery rate (FDR) of 5% using the Benjamini–Hochberg procedure [21]. The basic model was adjusted for age, sex, and energy intake. Model 1 was adjusted for age, sex, energy intake, PAL, and smoking. Fiber intake and BMI were separately added to model 1 to evaluate their role in the association. Their potential interactions with the exposure variables were also evaluated by introducing an interaction term to the regression. When both fiber intake and BMI were added to the regression, this constituted model 2. The *z* values from the negative binomial regressions of the trend over the three groups were used to create a heatmap. In sensitivity analysis we excluded users of antibiotics, defined as usage anytime during the last 6 months (*n* = 321 in added sugar analysis) and probiotics, defined as usage > 3 times per week (*n* = 477 in added sugar analysis).

Calculation of the Shannon diversity index (alpha diversity) and the Bray–Curtis dissimilarity index (beta diversity) were performed within the *vegan* package in R. To test for differences in beta diversity, we performed the permutation test Adonis in *vegan* adjusted for age and sex. The Shannon index and the log transformed Firmicutes:Bacteroidetes ratio were studied with multivariable linear regressions with adjustments in models 1 and 2, as described above. Differences in bacterial abundances between potential malabsorbers of fructose and the remaining study sample were examined using negative binomial regressions adjusted according to model 1.

## Results

As displayed in Table 1, high consumers of added sugar, SSBs, and ASBs were all younger compared to non- or low consumers. High consumption of SSBs was predominated by men, while high consumption of added sugar and ASBs tended to be predominated by women. High consumption of added sugar and SSBs were related to higher energy intake and E% of carbohydrates, but lower E% of fat and protein, while intake of ASBs did not relate to energy intake nor macronutrient distribution. High intake of added sugar, SSBs and ASBs were all related to low fiber intake. Only high consumption of ASBs was related to higher BMI. High intake of added sugar and SSBs was less common among those with a university degree, while such a relationship could not be seen for consumption of ASBs.

**Table 1:** Baseline characteristics of participants in the Malmö Offspring Study comparing low- and non-consumers with high consumers

	Added sugar		*P*	PC added × U-sugars		*P*	SSBs		*P*	ASBs		*P*
	*n* = 1371			*n* = 577			*n* = 1086			*n* = 1085		
	< 10%E	> 20E%		T1	T3		Non	High		Non	High	
*n*	458	136		193	192		314	347		669	147	
Mean (SD)												
Age, years	43.3 (13.5)	35.4 (12.7)	< 0.001	43.9 (12.4)	38.6 (12.8)	< 0.001	48.2 (12.1)	36.1 (12.8)	< 0.001	43.7 (13.7)	37.8 (12.3)	< 0.001
BMI, kg/m^2^	25.7 (4.1)	25.7 (5.6)	0.898	25.5 (4.0)	25.9 (5.2)	0.397	25.8 (4.6)	25.5 (4.8)	0.473	25.2 (4.2)	27.1 (5.2)	< 0.001
Waist circumference, cm	88.9 (12.3)	88.5 (14.9)	0.761	88.6 (12.3)	89.2 (13.9)	0.692	88.4 (13.6)	88.7 (13.1)	0.791	87.7 (12.3)	91.9 (14.5)	< 0.001
PAL	1.7 (0.13)	1.7 (0.12)	0.416	1.7 (0.14)	1.7 (0.14)	0.780	1.7 (0.13)	1.7 (0.14)	0.329	1.7 (0.13)	1.7 (0.15)	0.659
Energy, kcal/day	1858 (570)	2203 (688)	< 0.001	1910 (541)	2198 (664)	< 0.001	1770 (519)	2294 (689)	< 0.001	1995 (614)	2083 (703)	0.129
Carbohydrate, E%	41.0 (8.1)	52.4 (4.8)	< 0.001	41.2 (8.1)	49.6 (5.7)	< 0.001	43.3 (8.6)	47.5 (6.0)	< 0.001	45.0 (7.0)	44.6 (7.9)	0.474
Fat, E%	39.7 (7.6)	32.8 (4.7)	< 0.001	39.4 (7.6)	34.8 (5.3)	< 0.001	37.9 (7.9)	36.5 (5.5)	0.005	37.5 (6.3)	38.3 (7.4)	0.170
Protein, E%	19.3 (4.1)	14.8 (3.0)	< 0.001	19.5 (4.3)	15.6 (2.9)	< 0.001	18.8 (4.3)	16.1 (3.1)	< 0.001	17.4 (3.7)	17.1 (3.3)	0.294
Fiber intake, g/1000 kcal	10.6 (3.4)	7.4 (0.79)	< 0.001	10.7 (3.5)	8.6 (2.8)	< 0.001	11.1 (3.4)	8.3 (2.6)	< 0.001	10.1 (3.2)	8.7 (2.9)	< 0.001
Firmicutes:Bacteroidetes ratio*	0.09 (0.05)	0.28 (0.10)	0.079	0.14 (0.07)	0.41 (0.09)	0.017	0.02 (0.06)	0.34 (0.06)	< 0.001	0.15 (0.04)	0.30 (0.10)	0.142
Shannon index	2.2 (0.36)	2.2 (0.36)	0.314	2.2 (0.35)	2.3 (0.32)	0.033	2.2 (0.33)	2.3 (0.35)	0.223	2.2 (0.34)	2.2 (0.36)	0.876
%												
Women	52.2	56.6	0.363	52.3	61.5	0.071	71.7	44.1	< 0.001	56.2	57.1	0.835
University degree	45.2	35.1	0.041	48.7	38.4	0.043	47.4	33.1	< 0.001	41.6	44.8	0.484
Current smokers	12.2	14.7	0.447	8.3	10.9	0.378	9.6	14.7	0.044	10.6	15.7	0.083
Alcohol > 30 g/d	14.8	13.8	0.761	16.6	14.1	0.493	13.2	17.7	0.101	14.5	17.0	0.428
Antibiotics past 6 mo	14.7	9.7	0.176	11.7	12.9	0.747	10.7	13.1	0.371	12.1	13.6	0.628
Probiotics > 3 times/wk	6.2	5.9	0.921	10.2	8.9	0.709	10.2	11.0	0.757	9.5	11.9	0.387

*P* values are determined using *t* test for continuous variables and Chi-square tests to categorical variables. PC added × U-sugars is divided into equal tertiles. SSBs and ASBs are combinations of 4DFR and FFQ and are divided into three groups according to Fig. 1. *The Firmicutes:Bacteroidetes ratio is log transformed

Full lists of all 64 genera and their associations with the four different exposures in all regression models are displayed in Supplemental Tables 1, 2, 3, 4, 5, 6, 7, 8, 9, 10, 11, 12, 13, 14, 15, 16, 17, 18, 19, 20. As shown in Table 2, after adjusting for lifestyle factors in model 1, added sugar intake was found to be nominally positively associated with the *Streptococcus* genus and negatively associated with *Oxalobacter, Paraprevotella, Lachnobacterium, Odoribacter* and *Succiniclasticum*. After additionally adjusting for fiber intake and BMI, these genera remained nominally associated except for *Lachnobacterium*. However, none of these associations remained significant after FDR correction. In a subset of the study population (*n* = 577), in which the U-sugars was combined with added sugar intake using the PC method, *Odoribacter* and *Succiniclasticum* associated nominally negatively and *Lactobaccilus* associated nominally positively, with this composite measure after fiber and BMI adjustment, but did not hold after FDR correction.

**Table 2:** Adjusted means (SD) of normalized abundances over three groups of the exposures for those bacteria with *P*-trend < 0.05 over those three exposure groups

Model 1							Model 2						
Added sugar	< 10E%	10-20E%	> 20E%	ß-trend	*P*-trend	*P*’-trend	Added sugar	< 10E%	10-20E%	> 20E%	ß-trend	*P*-trend	*P*’-trend
*n* = 1371	455	780	136				*n* = 1371	455	780	136			
*Streptococcus*	7.72 (0.13)	8.08 (0.10)	8.66 (0.25)	0.05	0.001	0.060	*Streptococcus*	7.75 (0.14)	8.07 (0.10)	8.56 (0.26)	0.05	0.005	0.314
*Oxalobacter*	2.81 (0.19)	2.32 (0.12)	2.10 (0.28)	– 0.16	0.015	0.355	*Succiniclasticum*	0.19 (0.09)	0.06 (0.02)	0.06 (0.05)	– 0.81	0.029	0.518
*Paraprevotella*	4.03 (0.29)	3.19 (0.17)	3.21 (0.41)	– 0.16	0.019	0.355	*Paraprevotella*	4.00 (0.29)	3.19 (0.17)	3.29 (0.43)	–0.14	0.036	0.518
*Lachnobacterium*	4.13 (0.10)	4.03 (0.07)	3.60 (0.17)	–0.05	0.025	0.355	*Oxalobacter*	2.77 (0.19)	2.33 (0.12)	2.19 (0.30)	–0.14	0.038	0.518
*Odoribacter*	2.01 (0.16)	1.88 (0.11)	1.26 (0.19)	–0.17	0.033	0.355	*Odoribacter*	2.02 (0.16)	1.88 (0.11)	1.27 (0.20)	–0.17	0.040	0.518
*Succiniclastisum*	0.17 (0.07)	0.06 (0.02)	0.06 (0.05)	–0.74	0.033	0.355							

PC added × U-sugars	T1	T2	T3	ß-trend	*P*-trend	*P*’-trend	PC added × U-sugars	T1	T2	T3	ß-trend	*P*-trend	*P*’-trend
*n* = 577	193	192	192				*n* = 577	193	192	192			
*Odoribacter*	2.15 (0.24)	2.37 (0.26)	1.36 (0.16)	–0.22	0.012	0.507	*Odoribacter*	2.13 (0.24)	2.34 (0.26)	1.39 (0.17)	–0.21	0.020	0.822
*Streptococcus*	7.89 (0.21)	8.56 (0.21)	8.64 (0.22)	0.05	0.016	0.507	*Succiniclasticum*	0.26 (0.16)	0.11 (0.05)	0.05 (0.03)	–0.79	0.032	0.822
*Succiniclasticum*	0.25 (0.14)	0.11 (0.06)	0.05 (0.03)	–0.78	0.024	0.507	*Lactobacillus*	2.43 (0.19)	2.84 (0.21)	3.08 (0.24)	0.12	0.039	0.822
*Lactobacillus*	2.44 (0.19)	2.85 (0.21)	3.06 (0.23)	0.11	0.044	0.711							

SSBs	Non	Medium	High	ß-trend	*P*-trend	*P*’-trend	SSBs	Non	Medium	High	ß-trend	*P*-trend	*P*’-trend
*n *= 1086	314	425	347				*n* = 1086	314	425	347			
*Lachnobacterium*	4.23 (0.13)	4.12 (0.10)	3.61 (0.11)	–0.08	0.0003*	0.020	*Lachnobacterium*	4.19 (0.13)	4.11 (0.10)	3.65 (0.11)	–0.07	0.002	0.088
*Dialister*	8.04 (0.28)	9.05 (0.25)	9.35 (0.30)	0.07	0.003	0.090	*Dialister*	8.04 (0.30)	9.05 (0.25)	9.35 (0.30)	0.07	0.003	0.088
*Lactobacillus*	2.36 (0.15)	2.74 (0.14)	3.04 (0.19)	0.13	0.007	0.111	*Lactobacillus*	2.33 (0.15)	2.73 (0.14)	3.09 (0.19)	0.14	0.004	0.088
[*Eubacterium*]	5.12 (0.21)	5.53 (0.18)	6.00 (0.23)	0.08	0.007	0.111	*Cetobacterium*	0.16 (0.27)	0.02 (0.02)	0.004 (0.004)	–1.84	0.044	0.607
*Roseburia*	9.20 (0.18)	9.16 (0.15)	8.58 (0.17)	–0.04	0.016	0.208							
*Anaerotruncus*	2.16 (0.13)	2.48 (0.12)	2.66 (0.15)	0.10	0.021	0.222							
Unknown genus in the Peptostreptococceae family	6.20 (0.15)	6.56 (0.13)	6.68 (0.15)	0.04	0.039	0.353							

ASBs	Non	Medium	High	ß-trend	*P*-trend	*P*’-trend	ASBs	Non	Medium	High	ß-trend	*P*-trend	*P*’-trend
*n* = 1085	669	269	147				*n* = 1085	669	269	147			
*Prevotella*	6.01 (0.17)	6.82 (0.29)	7.20 (0.42)	0.10	0.001	0.075	*Prevotella*	6.04 (0.17)	6.78 (0.29)	7.12 (0.42)	0.09	0.004	0.257
Unknown genus in the RF16 family	1.81 (0.12)	2.32 (0.24)	2.56 (0.35)	0.19	0.007	0.234	Unknown genus in the RF16 family	1.82 (0.12)	2.30 (0.23)	2.50 (0.34)	0.18	0.017	0.525
*Sutterella*	3.49 (0.15)	4.44 (0.31)	4.16 (0.39)	0.12	0.013	0.265	*Sutterella*	3.51 (0.16)	4.40 (0.30)	4.12 (0.39)	0.11	0.025	0.525
Unknown genus in the SHA98 order	2.78 (0.15)	2.40 (0.22)	2.06 (0.25)	–0.15	0.017	0.265	*Lachnospira*	5.86 (0.10)	5.67 (0.15)	5.40 (0.20)	–0.04	0.037	0.588
[*Eubacterium*]	5.32 (0.14)	5.99 (0.24)	5.90 (0.33)	0.07	0.021	0.268							
Unknown genus in the Christensenellaceae family	4.31 (0.10)	4.21 (0.15)	3.79 (0.19)	–0.05	0.032	0.296							
*Lachnospira*	5.86 (0.09)	5.68 (0.15)	5.40 (0.19)	–0.04	0.032	0.296							

PC added × U-sugars is divided into equal tertiles. SSBs and ASBs are combinations of 4DFR and FFQ and are divided into three groups according to Fig. 1. Negative binomial regressions were used. Model 1 is adjusted for age, sex, energy intake, smoking and PAL. Model 2 is additionally adjusted for fiber intake and BMI

*Holds after FDR correction. *P*’ is adjusted for FDR using the Benjamini–Hochberg method

SSB intake was nominally positively associated with *Dialister*, *Lactobacillus, [Eubacterium], Anaerotruncus* and an unknown genus in the Peptostreptococcaceae family and nominally negatively associated with *Lachnobacterium* and *Roseburia* in model 1. After further adjustment for both fiber intake and BMI, *Lachnobacterium, Dialister,* and *Lactobacillus* remained nominally associated. After FDR correction, only *Lachnobacterium* remained negatively and significantly associated with SSB intake until fiber intake was added as a covariate to the model; however, *Lachnobacterium* remained the genera with the strongest association with SSB intake. BMI adjustment did not markedly influence this association, nor did exclusion of participants that reported regular consumption of probiotics (> 3 times/week) or had taken antibiotics at any time point during the last 6 months (Supplemental Table 12).

**Fig. 2:**
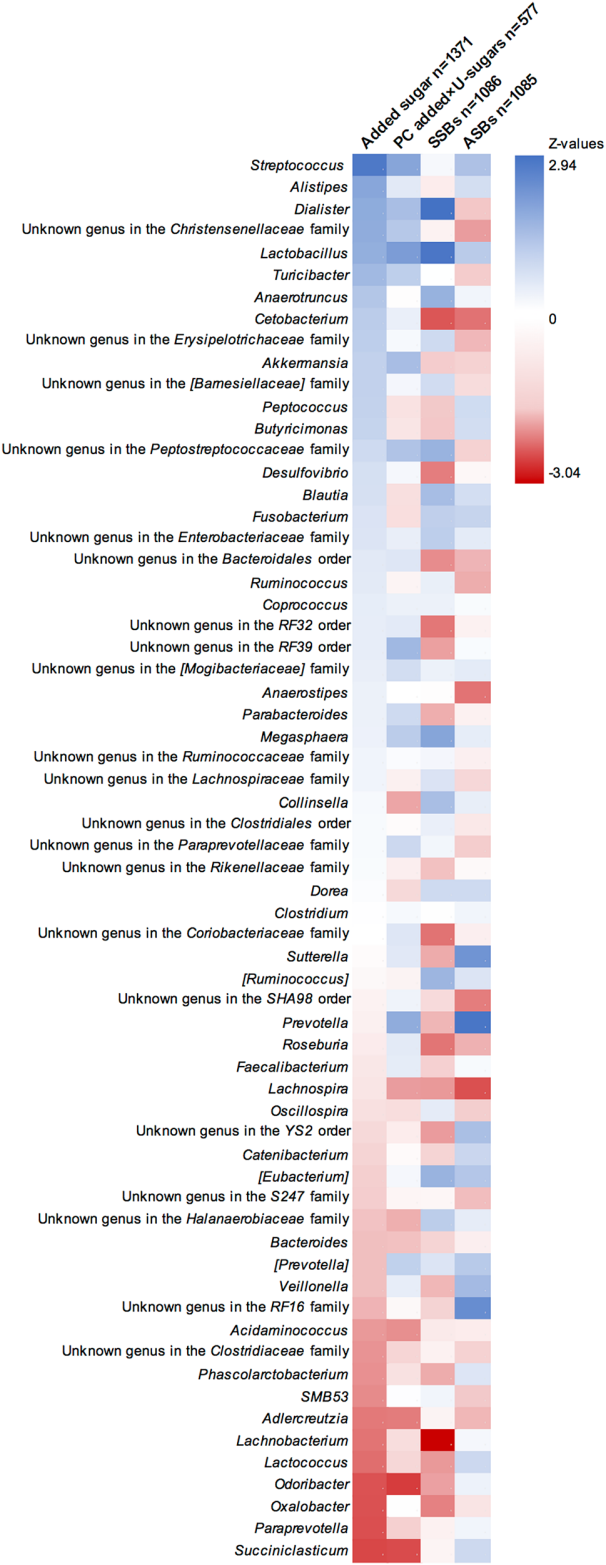
Heatmap of *z* values from trend over three groups of exposure using negative binomial regressions adjusted according to model 2 (age, sex, energy intake, smoking, PAL, fiber intake, and BMI). Genera are sorted according to the *z* value from regressions with added sugar intake

*Prevotella, Sutterella, [Eubacterium]* and an unknown genus in the RF16 family were nominally positively associated with ASB intake after model 1 adjustment and *Lachnospira* and two unknown genera in the Christensenellaceae family and SHA98 order were found to be nominally negatively associated. After additional adjustment of fiber intake and BMI, *Prevotella, Sutterella, Lachnospira,* and an unknown genus in the RF16 family remained nominally associated. None of these associations remained after FDR correction. A summary of all associations with all 64 genera adjusted according to model 2 is displayed in the heatmap (Fig. 2).

No effect modification was observed for fiber intake or BMI on any association with added sugar. For the other exposure variables, a few associations showed nominally significant interaction with either fiber intake or BMI (none remained after FDR correction), but not among any of the significantly associated bacterial genera (Supplemental Tables 2, 7, 12 and 17). After excluding participants reporting any use of antibiotics the past 6 months, the associations between both added sugar and the PC of added sugar × U-sugars and abundance of *Succiniclasticum* were weakened, while strengthened after exclusion of regular probiotic users. No other clear patterns could be observed from the results of the sensitivity analysis (Supplemental Tables 2, 5, 7, 10, 12, 15, 17, and 20).

As presented in Table 3, we observed a significant positive association between added sugar intake, the PC added × U-sugars and SSB intake and the Firmicutes:Bacteroidetes ratio in model 1, but only the association with SSB intake remained after adjustment for fiber intake and BMI (*P* = 0.048). We observed no association between ASB intake and the Firmicutes:Bacteroidetes ratio. In addition, no associations between added sugar intake, SSB intake or ASB with the Shannon index were observed, but the PC added × U-sugars associated positively until fiber intake and BMI were added as covariates. Further, significant differences in beta diversity were observed between the three intake groups for added sugar (R^2^ = 0.003, *P* < 0.001), SSBs (*R*^2^ = 0.007, *P* < 0.001), and ASBs (*R*^2^ = 0.004, *P* < 0.001). Differences in beta diversity between tertiles of PC added × U-sugars were *R*^2^ = 0.002, *P* = 0.09.

**Table 3:** Associations between three categories of added sugar intake, the PC of added × U-sugars, SSB intake and ASB intake and the Firmicutes:Bacteroidetes ratio and the Shannon index

	Added sugar *n* = 1371		PC added × U-sugars *n* = 577		SSBs *n* = 1086		ASBs *n *= 1085	
	ß	*P*	ß	*P*	ß	*P*	ß	*P*
Firmicutes:Bacteroidetes ratio*								
Model 1	0.119	0.021	0.117	0.041	0.108	0.021	0.049	0.291
Model 2	0.098	0.059	0.089	0.120	0.094	0.048	– 0.008	0.864
Shannon index								
Model 1	0.026	0.087	0.037	0.031	0.019	0.187	0.005	0.713
Model 2	0.019	0.213	0.031	0.080	0.014	0.352	– 0.005	0.743

Determined using linear regression. Model 1 is adjusted for age, sex, energy intake, smoking and PAL. Model 2 is additionally adjusted for fiber intake and BMI

*Firmicutes:Bacteroidetes ratio is log transformed

Among those who reported high intake of added sugar but had low urinary fructose (i.e., potential malabsorbers of fructose), *[Eubacterium]* (*P* = 0.012)*, Prevotella* (*P* = 0.054) and *Megasphaera* (*P* = 0.059) were the top three genera (based on *P* value) which were higher abundant in comparison to the remaining study sample after adjustment in model 1*,* but none of these remained significant after correction for FDR (Supplemental Fig. 1).

## Discussion

In this cross-sectional analysis of Swedish adults, using two different dietary assessments methods and the overnight urinary sugars biomarker for improved intake measurements, we observed various genera that nominally associated with intake of added sugar, SSBs, and ASBs. However, only *Lachnobacterium* was significantly inversely associated with SSB intake after FDR correction, whereas no other association remained significant. Furthermore, we observed a significant positive association between SSB intake and the Firmicutes:Bacteroidetes ratio after adjusting for both fiber intake and BMI.

This is the largest epidemiological study that has investigated the associations between sugar intake and the gut microbiota. To the best of our knowledge, the only other observational study that has investigated this potential link with individual gut bacteria did so only for fructose intake and in a limited sample of 52 obese American teenagers [22]. In contrast to that study, which observed a negative association between fructose intake and abundance of *Streptococcus* and [*Eubacterium*], we here observed a positive association between added sugar intake and the *Streptococcus* genera and positive associations between intake of both SSBs and ASBs and [*Eubacterium*] (not after FDR correction). However, our study sample is more than 25 times larger and of varying ages and BMI. In another observational study of 1135 participants from the Netherlands, with stool samples analyzed using shotgun metagenomic sequencing, intake of SSBs, but not ASBs, was associated with lower microbial alpha diversity, while associations with individual bacteria were not reported [23]. An association between SSB intake and alpha diversity could not be replicated in the present study where 16S sequencing was used.

Our findings regarding intake of SSBs also agree with previous findings in MOS where [*Eubacterium*] and *Anearotruncus* were negatively associated with a data-driven health-conscious food pattern by PC analysis, partly represented by low intake of SSBs, while *Roseburia* was positively associated with this health-conscious food pattern [20]. Intake of added sugar and SSBs could in general be considered a marker for unhealthy lifestyle and a diet low in fiber. As commonly observed, fiber intake was lower in high consumers of both added sugar, SSBs and ASBs as compared to low- or non-consumers in our study. The results after adjustment for fiber intake indicates a role as a confounder. Nevertheless, fiber intake was not found to be a significant effect modifier in our analyses. Regarding *Lachobacterium,* that was observed to associate significantly with SSB intake, this genus is very limitedly studied and has never been associated with any cardiometabolic traits.

Although the knowledge is limited regarding the links between intake of sugar and the gut microbiota, we know that there is a link between sugar intake and obesity [3] and a potential link between obesity and the gut microbiota [24]. It has been suggested, but also questioned, that obese individuals may have less rich and diverse microbiota composition and that their proportion of Firmicutes may be higher, while Bacteroidetes may be lower [24–26]. We observed a significant positive association between SSB intake and the Firmicutes:Bacteroidetes ratio, even after adjustment for fiber intake and BMI. We saw no association between ASB intake and the Firmicutes:Bacteroidetes ratio, despite that only intake of ASBs was related to higher BMI and waist circumference, which, however, likely is not an association due to a causality, but rather reversed, because people tend to change to ASBs instead of SSBs when experiencing weight and health problems. Regarding diversity, we could see no differences in alpha diversity (Shannon index), but statistically significant differences in beta diversity. However, the R^2^ values were rather low and the differences are therefore likely to be clinically insignificant.

Fructose malabsorption is a hypothesis of how a high sugar intake might affect the gut microbiota composition. In a speculative attempt to evaluate this, we here demonstrate a novel potential application for the urinary fructose measurement. However, we cannot ascertain the accuracy of this method. It may be, that those who are truly not absorbing fructose, are in fact those in which we cannot even detect fructose in the urine and hence do not have a valid urinary fructose measurement. Out of the 824 analyzed urine samples in this study sample of MOS (*n* = 1371), 584 had a valid fructose measurement within the calibration range. There are likely also more factors that could influence the amount of fructose urinarily excreted other than the amount absorbed in the small intestine [27]. Additionally, simultaneous intake of glucose enhances fructose absorption, i.e. fructose absorption is more efficient when consuming sucrose than free fructose [11]. In Sweden, sucrose is used for sweetening and usage of high-fructose corn syrup or other free fructose is very rare. Hence, fructose malabsorption may not be as frequent in this population as in countries where high-fructose corn syrup is used. Furthermore, fructose is often fermented early in the colon, or even at the end of the small intestine, so the bacteria involved in this process may not be present to the same degree in the distal colon or in the feces, which is from where our samples have been obtained [11]. For example, *Streptococcus* has been observed to be important for small-intestinal fermentation of sugars [28]. As our study is limited to fecal samples, examination of any potential link between added sugar intake and so-called small intestinal bacterial overgrowth, was not possible [29].

In addition to compositional changes as an effect of unabsorbed sugars or sweeteners, Di Rienzi et al. describes in a recent review [7] how intake of sugar and sweeteners could alter the gut microbiota by transcriptional changes, e.g. as in the case of suppression of the protein involved in the colonization ability of *Bacteroides thetaiotaomicron* seen in mice [13]*,* or by genetic adaptations occurring within bacterial strains [7]. It is unfortunate that *Bacteroides thetaiotaomicron* is not measured on species level in our sample, as it has been linked to sugar intake in the previous literature [13]. However, we have data on the *Bacteroides* genus, in which around 100 species are known [30]*. Bacteroides* showed non-significant negative associations with added sugar, PC added × U-sugars and SSBs as seen in Fig. 2. Hence, we need metagenomic sequencing for evaluating these associations on both species and strain level to properly consider the different potential pathways in which sugars might affect the gut microbiota as suggested by Di Rienzi et al. (compositional, transcriptional and genetic).

We were limited to the assessment of low-calorie/nonnutritive sweeteners intake solely from beverages (ASB intake), which does not cover the total, but the majority, of the intake of low-calorie/nonnutritive sweeteners [31]. The most common low-calorie/nonnutritive sweeteners used in Sweden are aspartame and acesulfame-K, which both are fairly well-absorbed in the small intestine [7], but have only been studied in relation to gut microbial changes in rodents and not in humans [8]. Sucralose, on the other hand (which is moderately used in Sweden), is only absorbed to around 10–30% and has been observed to cause alterations of gut microbiota composition in rodents [32, 33]. As for saccharin, in addition to rodent studies, only one human trial of limited size has been published, showing glucose intolerance explained by gut microbial shifts with high consumption [17]. However, saccharin is only marginally used in Sweden and do thus probably not explain much of the associations observed in our study.

Another main limitation of this study is the single time point for measurements of both the diet and fecal microbiota, which limits this to a cross-sectional comparison without possibility to study causation. Additionally, current understanding of confounders in the gut microbiota analyses is limited, making residual confounding very plausible. Furthermore, the distribution of some bacterial abundances is highly skewed and some of the observed associations should therefore be interpreted with caution even though negative binomial regressions were used to deal with the varying quality of the distributions. This particularly includes *Succiniclasticum* and *Cetobacterium* among the genera observed to be significantly associated. It is, however, an important strength that we have considered both short- and long-term assessment of SSB and ASB intake by combining intake data from the 4DFR and the FFQ and used the support of the overnight urinary sugar biomarker.

In conclusion, many previous studies have discussed how intake of added sugar and sweetened beverages may increase cardiometabolic risk and our study can only find very modest support for that such risk could be partially acting through mechanisms involving the gut microbiota. After full covariate adjustment, we found an association between SSB intake and the Firmicutes:Bacteroidetes ratio, which previously has been linked to obesity. Among the 64 individual bacterial genera, only the inverse association between SSB intake and *Lachnobacterium* remained significant after adjustment of multiple testing. Both epidemiological and interventional studies, preferably with metagenomic sequencing and of larger study samples, are needed to further evaluate if a link exists between intake of sugars and sweeteners and the human gut microbiota.

## Electronic supplementary material

Below is the link to the electronic supplementary material.

Supplementary file1 (XLSX 540 kb)

## Data Availability

The dataset for this manuscript cannot be made publicly available because of ethical and legal restrictions. Requests to access the dataset should be directed to the Chair of the Steering Committee for the Malmö cohorts, see instructions at https://www.malmo-kohorter.lu.se/english.

## Abbreviations

4DFR: 4 Day food record
ASB: Artificially sweetened beverage
BMI: Body mass index
FDR: False discovery rate
FFQ: Food frequency questionnaire
MOS: Malmö offspring study
PAL: Physical activity level
PC: Principal component
SSB: Sugar-sweetened beverage
U-sugars: Sum of urinary sucrose and fructose adjusted for urine osmolality
